# A model of atherosclerosis using nicotine with balloon overdilation in a porcine

**DOI:** 10.1038/s41598-021-93229-1

**Published:** 2021-07-01

**Authors:** Munki Kim, Han Byul Kim, Dae Sung Park, Kyung Hoon Cho, Dae Young Hyun, Hae Jin Kee, Young Joon Hong, Myung Ho Jeong

**Affiliations:** 1 The Cardiovascular Convergence Research Center of Chonnam, National University Hospital Designated by Korea Ministry of Health and Welfare, Gwangju, 61469 Republic of Korea; 2 Korea Cardiovascular Stent Research Institute, Jangsung, 57248 Republic of Korea; 3 Division of Cardiology of Chonnam, National University Hospital, Cardiovascular Convergence Research Center Nominated By Korea Ministry of Health and Welfare, Gwangju, Republic of Korea

**Keywords:** Cardiology, Cardiovascular biology

## Abstract

Pigs are important experimental animals for cardiovascular research. Few porcine coronary atherosclerosis models have been developed; however, their induction requires more than six months. We developed a porcine coronary artery atherosclerosis model using nicotine injection with a balloon overdilation. A coronary balloon was placed in the porcine coronary artery and overdilated to induce a mechanical injury. Nicotine was administrated via intramuscular injection every day, and changes in the coronary artery were observed after four weeks. Coronary angiography revealed nicotine injection with a balloon overdilation group showed narrowing of the coronary artery at the injury site. The combination of balloon and nicotine significantly increased the intimal hyperplasia in optical coherence tomography analysis. Proliferated tunica media were noted in the nicotine injection with balloon overdilation groups and lack of collagen was observed in the tunica media at eight weeks. Quantitative analysis showed increased smooth muscle actin alpha (SMA), cluster of differentiation 68 (CD68), and Krüppel-like factor 4 (KLF4) in the nicotine injection with balloon overdilation groups. Immunohistochemistry results showed CD68-positive cells displayed SMA- and KLF4-positive reactivity in the border zone of the intimal hyperplasia. Our results show that nicotine injection with balloon overdilation can induce atherosclerotic lesions within one month, which can serve as an alternative pig animal model for the development of coronary stents.

## Introduction

Atherosclerosis is a chronic progressive inflammatory disease. It is characterized by a large number of macrophages, a necrotic core containing rich lipids, and cellular debris^1,2^. As the disease progress, the phenotype of smooth muscle cells (SMCs), the primary cell of the vessel wall intima, changes in terms of proliferation, and loss of contractility, increase synthesis, and reduce the expression of SMC biomarkers^3,4^. The precise reasons for the rupture or erosion of an unstable atherosclerosis remain unknown; arthrosclerosis lesions can rupture and induce thrombotic events that lead to myocardial infarction or stroke, the major cause of human death in worldwide^5^.

Nicotine is the main ingredient of cigarettes and smoking is the single most important risk factor for tumorigenesis, cardiovascular disease, and peripheral vascular diseases^6,7^. Nicotine has been shown to change the proliferation of endothelial cells and enhance angiogenesis^8–10^. It also activates atherosclerosis associated factors in endothelial cells, such as, endothelial nitric oxide synthase, tissue-type plasminogen activator, platelet-derived growth factor, and basic fibroblast growth factor In the presence of vascular disease^11,12^. It also changes endothelial cell morphology, induces inflammatory responses within endothelial cells and increases endothelial cell death^10,13^.

Pigs have been studied as models of human cardiovascular diseases and have been used to validate cardiovascular medical devices, especially coronary stents. Their cardiovascular systems, artery distribution, and heart size are similar to those in humans^14^. They spontaneously develop coronary artery atherosclerosis because of their lipid profiles^15,16^. Despite these advantages, the use of pigs as experimental animal models has been restricted. Complications related to the induction of atherosclerosis also create restrictions. Further, a series of studies have demonstrated a variety of atherosclerosis porcine models, but the induction of atherosclerotic lesions requires more than six months^17,18^.

This study aimed to develop an atherosclerosis porcine model using nicotine injection and balloon overdilation.

## Results

### Optical coherence tomography analysis

**Figure 1:**
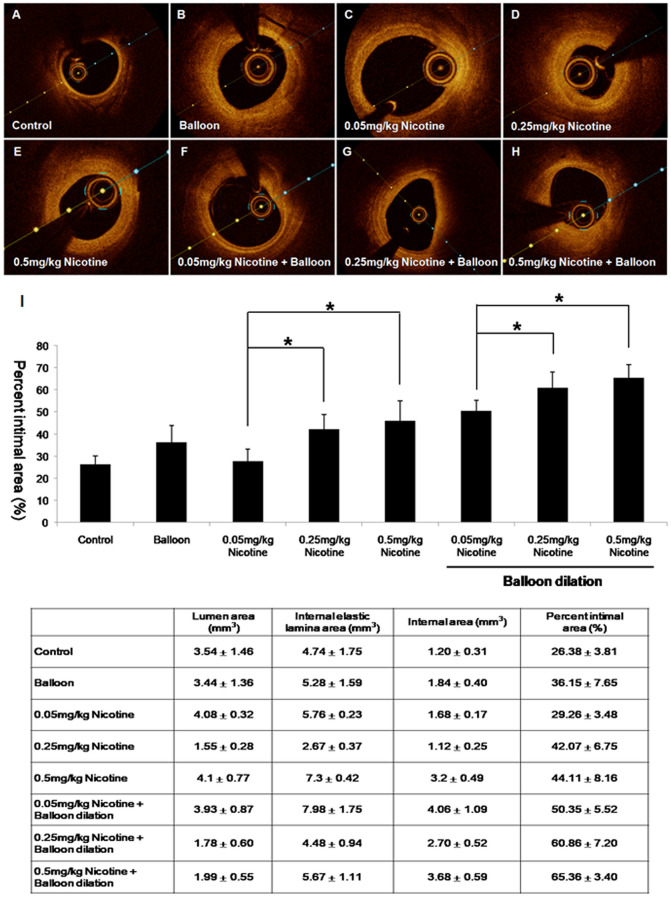
Intimal hyperplasia after balloon overdilation and nicotine injection in the coronary arteries. The control group showed thin and compact coronary artery wells (**A**). All treatment groups showed an increase of the percentage intimal hyperplasia in the OCT images (**B**–**H**). In the volumetric analysis of OCT, percentage intimal area significantly increased in the balloon overdilation group compared to the control groups (26.38 ± 3.81% in the control group vs 36.15 ± 7.65 in the balloon overdilation groups vs 29.26 ± 3.48 in the 0.05 mg/kg nicotine injection group). The percentage intimal area significantly increased in the 0.25 mg/kg and 0.5 mg/kg nicotine injection groups compared to the 0.05 mg/kg nicotine injection group (29.26 ± 3.48 in the 0.05 mg/kg nicotine injection group vs 42.07 ± 6.75 in the 0.25 mg/kg nicotine injection group vs 44.11 ± 8.16 in the 0.5 mg/kg nicotine injection group). The percentage intima area significantly increased in the 0.25 mg/kg nicotine with balloon overdilation and 0.5 mg/kg nicotine with balloon overdilation groups compared to the 0.05 mg/kg nicotine injection with balloon overdilation group (50.6 ± 5.52 in the 0.05 mg/kg nicotine injection with balloon overdilation vs 60.86 ± 7.2 in the 0.25 mg/kg nicotine injection with balloon overdilation vs 65.36 ± 3.40 in the 0.5 mg/kg nicotine injection with balloon overdilation groups; *p* < 0.05) (**I**).

Optical coherence tomography (OCT) analysis was performed to identify changes in the intima of the coronary artery. The control group showed thin and compact coronary artery walls (Fig. 1A). All treatment groups showed increase of the percent intimal hyperplasia in the OCT images (Fig. 1B–H). In the volumetric analysis of OCT, percent intimal area significantly increased in the balloon overdilation group compared to that in the control group (26.38 ± 3.81% in the control group vs 36.15 ± 7.65 in the balloon overdilation groups vs 29.26 ± 3.48 in the 0.05 mg/kg nicotine injection group). The percent intimal area significantly increased in the 0.25 mg/kg and 0.5 mg/kg nicotine injection groups compared to the 0.05 mg/kg nicotine injection group (29.26 ± 3.48 in 0.05 mg/kg in the nicotine injection group vs 42.07 ± 6.75 in the 0.25 mg/kg nicotine injection group vs 44.11 ± 8.16 in the 0.5 mg/kg nicotine injection group). The percent intimal area significantly increased in the 0.25 mg/kg nicotine with balloon overdilation and 0.5 mg/kg nicotine with balloon overdilation groups compared to the 0.05 mg/kg nicotine injection with balloon overdilation group (50.6 ± 5.52 in the 0.05 mg/kg nicotine injection with balloon overdilation vs 60.86 ± 7.2 in the 0.25 mg/kg nicotine injection with balloon overdilation vs 65.36 ± 3.40 in the 0.5 mg/kg nicotine injection with balloon overdilation) (Fig. 1I).

### Histopathologic findings after nicotine injection with balloon overdilation

**Figure 2:**
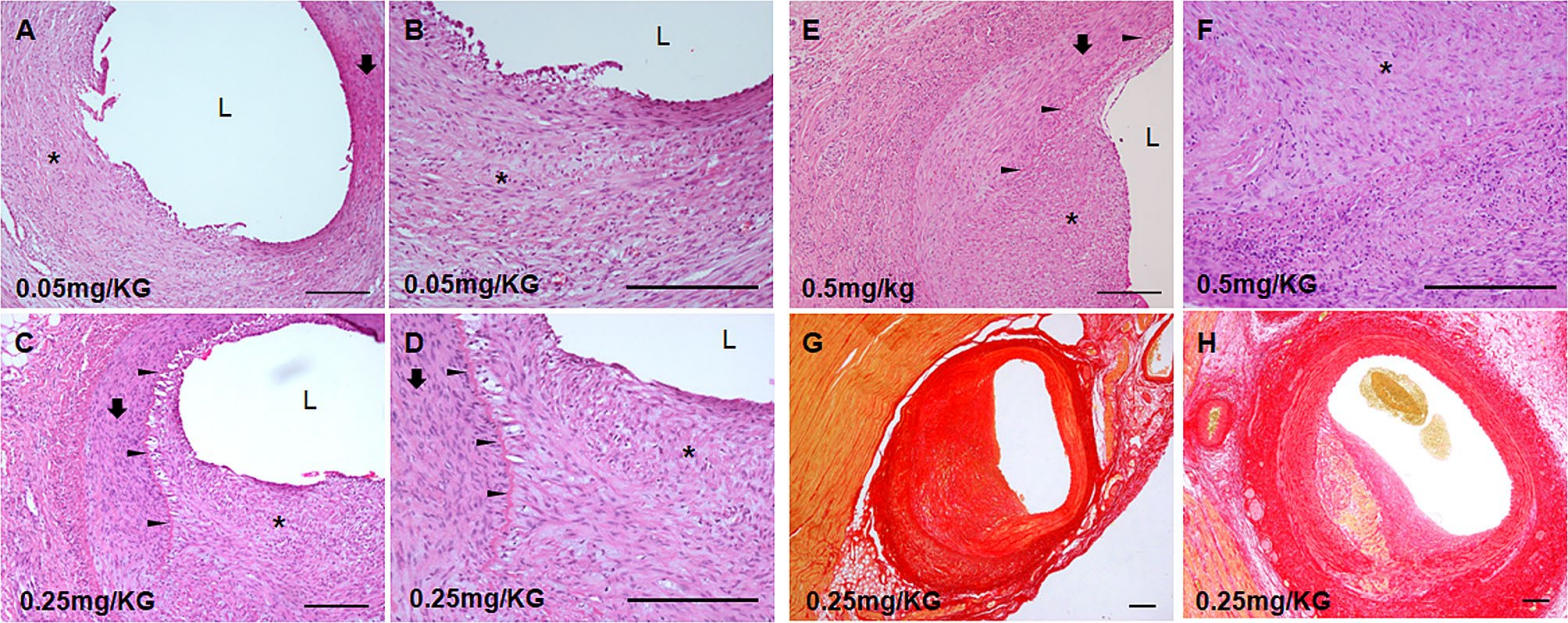
Hyperplasia of the coronary arteries after nicotine injection with balloon overdilation. (**A**,**B**) The dose of 0.05 mg/kg nicotine with balloon overdilation delayed the healing of the injured internal elastic lamina and induced the proliferation of the tunica media. (**C**,**D**) The dose of 0.25 mg/kg nicotine injection with balloon overdilation induced clear intimal hyperplasia compared to that seen in the lower dose and simple balloon overdilation groups. (**E**,**F**) The dose of 0.5 mg/kg nicotine injection with balloon overdilation induced intimal hyperplasia similar to the 0.25 mg/kg nicotine injection with balloon overdilation. (**G**) Picrosirius red staining results showed collagen positive intimal hyperplasia in the 0.25 mg/kg nicotine with balloon overdilation groups at 4 weeks. (**H**) Lack of collagen positive was observed in the 0.25 mg/kg nicotine with balloon overdialtion groups at 8 weeks. *L* lumen, arrow head: internal elastic lamina, arrow: tunica media, star: intimal hyperplasia. Bar: 100 µm.

Each dose of nicotine was administrated to the pigs whose coronary arteries were damaged by coronary balloon inflation to achieve intimal hyperplasia of the coronary arteries. Unlike simple nicotine injection or balloon overdilation, 0.05 mg/kg nicotine injection with balloon overdilation induced intimal hyperplasia (Fig. 2A,B). The 0.25 mg/kg nicotine injection with balloon overdilation showed intimal hyperplasia of the tunica media covering the internal elastic membrane (Fig. 2C,D). 0.5 mg/kg nicotine injection with balloon overdilation group exhibited similar intimal hyperplasia as that of the 0.25 mg/kg nicotine groups (Fig. 2E,F). The area of intimal hyperplasia significantly increased in the 0.25 mg/kg and 0.5 mg/kg nicotine injection with balloon overdilation groups compared to the 0.05 mg/kg nicotine injection with balloon overdilation groups (Supplemnetary Fig. S2). Picrosirius red staining showed collagen positive intimal hyperplasia in the 0.25 mg/kg nicotine injection with balloon overdialation groups at 4 weeks. Lack of the collagen was observed in the intimal hyperplasia in the 0.25 mg/kg nicotine injection with balloon overdialation group at 8 weeks (Fig. 2G,H).

### The expression of KLF4 and CD68 expression in the coronary arteries

**Figure 3:**
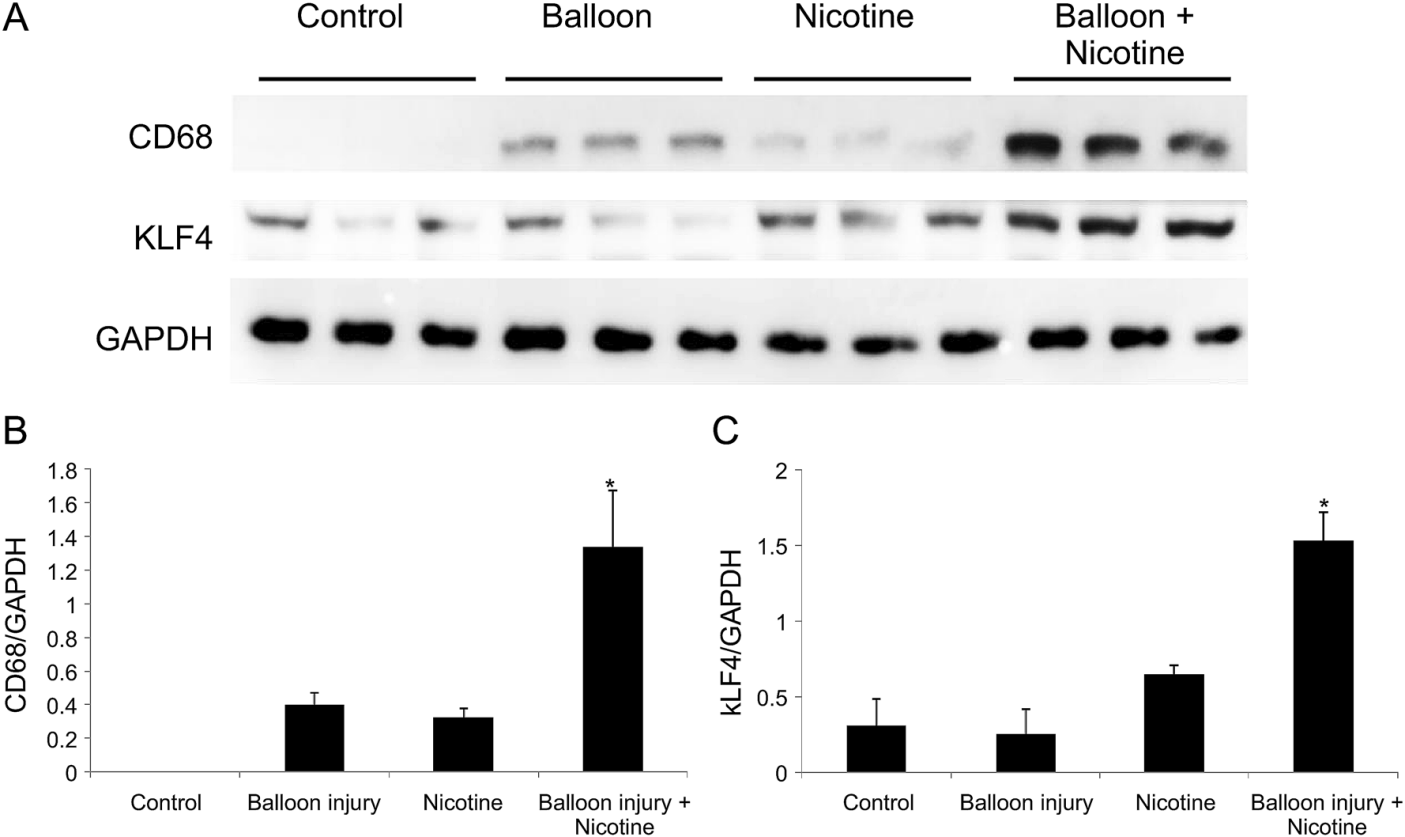
Increased expression of Krüppel-like factor 4 (KLF4) and cluster of differentiation 68 (CD68) in the nicotine injection with balloon overdilation groups. (**A**) Western blot analysis was performed to identify the changes in KLF4 and CD68 expression. (**B**) In the control groups, CD68 was not expressed in the coronary arteries. Balloon overdilation or nicotine injection increased CD68 expression. The nicotine injection with balloon overdilation groups had significantly increased CD68 expression compared to the other groups. (**C**) KLF4 expression was similar in the control and balloon overdilation groups and nicotine injection increased KLF4 expression. Nicotine injection with balloon overdilation significantly increased KLF4 expression in the coronary arteries. *P < 0.05.

Western blot analysis of the coronary artery samples was performed to quantify changes in KLF4 and CD68 expression (Fig. 3A). Samples from the control group did not express CD68, whereas those from the nicotine injection with balloon overdilation groups showed significantly increased CD68 expression (Fig. 3B, Supplementary Fig. S3). Similar KLF4 expression was observed in the control and balloon overdilation groups, and nicotine injection increased the KLF4 expression. However, the nicotine injection with balloon overdilation group had significantly increased KLF4 expression compared to the other groups (Fig. 3C).

### Intimal hyperplasia via KLF4 after nicotine injection with balloon overdilation

**Figure 4:**
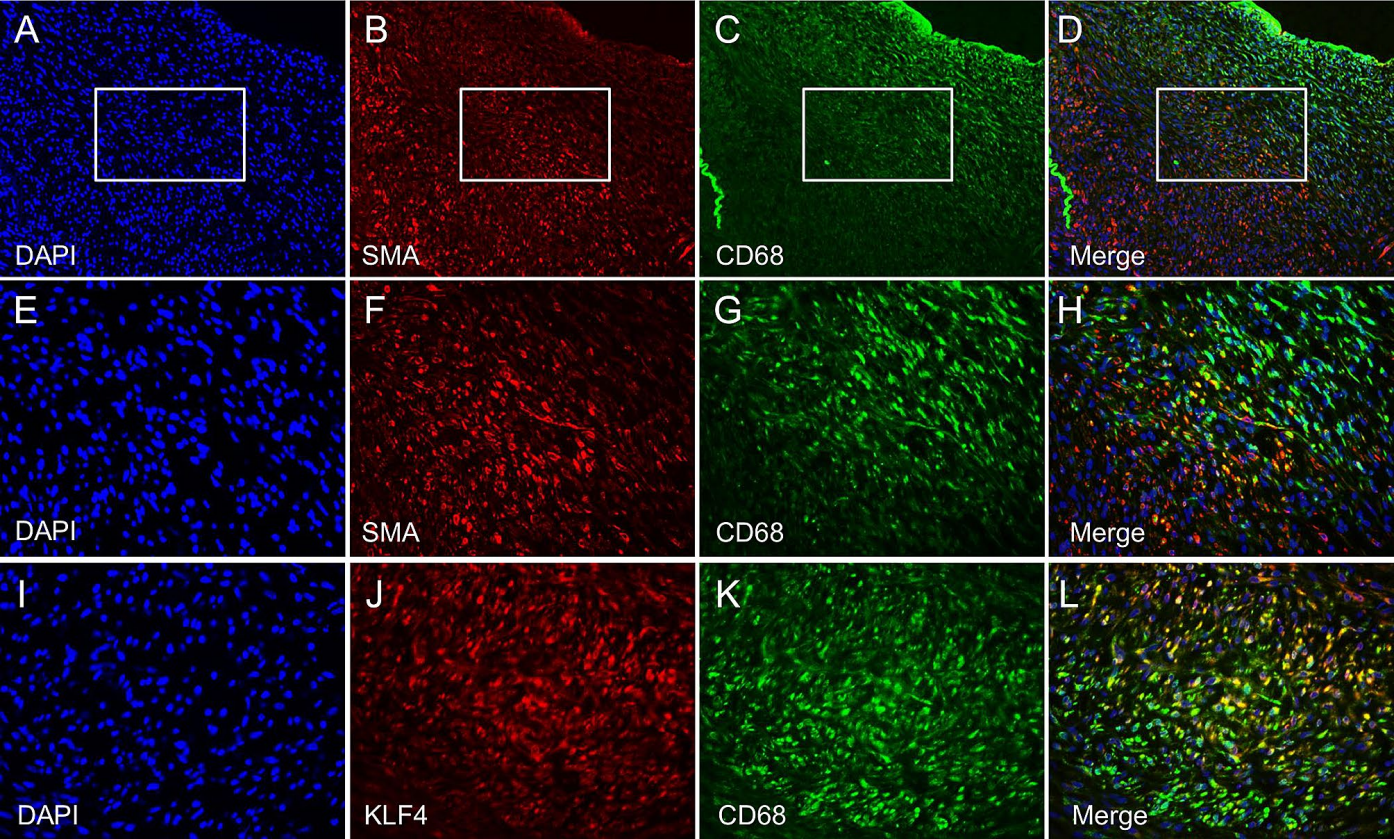
Alpha smooth muscle actin (SMA) positive cells and cluster of differentiation 68 (CD68) in the intimal hyperplasia lesion of nicotine injection with balloon overdilation group. (**A**–**D**) In the hyperplastic lesion of nicotine injection with balloon overdilation groups, SMA positive cells and CD68 positive cells were separately observed. (**E**–**H**) An examination at higher magnification, board zone revealed SMA and CD68 co positive cells. (**I**–**L**) CD68 positive cells co expressed KLF4.

In the nicotine injection with balloon overdilation groups, immunohistochemical analysis was performed to identify the expression of SMA, CD68, and KLF4. In hyperplastic lesions, SMA-positive reactive cells and CD68 positive cells were observed separately (Fig. 4A–D). An examination at a higher magnification board zone revealed SMA and CD68 co-positive cells (Fig. 4E–H). Moreover, the CD68 positive cells co-expressed KLF4 (Fig. 4I–L).

## Discussion

According to our experiments, the nicotine injection with balloon overdilation is a novel approach for inducing porcine coronary atherosclerosis. This animal model can serve as a suitable pathological model for future coronary stent development in atherosclerosis treatment.

Several small and large animal models of atherosclerosis have been developed in the field of research. Although small animal models, such as those of mice, rats, and rabbits, have provided valuable information about cardiovascular diseases, there are limitations to the clinical application of these animal models. For example, mouse atherosclerotic models are relatively inexpensive and feature easy breeding and genetic manipulation compared to large animal models. However, these models naturally develop atherosclerotic lesions in the aortic root, not in the coronary arteries, and do not develop thick fibrous cap atherosclerotic lesions^1,20^. Small size of the model is another key limitation. Their size restricts the use of the model in the development of human coronary devices (such as coronary stents) and the use of imaging techniques or tools. Pigs are standard experimental animals for evaluating a coronary stent, and pig atherosclerosis models have been developed. However, previous porcine models are limited in their ability to be used in coronary stent research, including extended time for lesion induction, various severities of lesions, and unpredictable plaque locations^14,21,22^. Our model can appoint appropriate lesions of the coronary arteries, and the severity of the lesion is relatively uniform. These features can serve as an alternative pig animal model for research on coronary stents.

Balloon overdilation induced luminal stenosis in the porcine coronary artery. Percutaneous coronary intervention was performed to open blocked or stented coronary arteries. During this process, mechanical stimulation of the artery is inevitable, and an experimental study was conducted to identify the response of the injured coronary artery to balloon overdilation. Balloon overdilation mechanically damages the coronary artery and induces endothelial denudation. Injured arteries expose the blood and induce thrombus formation and inflammatory response. Consequently, the injured arteries formed stenotic lesions^23,24^. Our results are consistent with those of the previous studies. Balloon-injured porcine coronary arteries demonstrated damage to arterial wall and luminal stenosis.

Nicotine injection can induce the proliferation of endothelial cells within coronary arteries. The coronary arteries consist of the tunica intima, media, and adventitia, and the tunica intima is covered with vascular smooth muscle cells (VSMCs), which express the transmembrane ligand-gated ion channels nicotinic acetylcholine receptors^15,25^. Nicotine is the major hazardous component of cigarettes, and a series of studies have demonstrated the atherogenic effect of nicotine. Nicotine exposure disturbs the normal expression of endothelial cell-derived platelet-derived growth factor, basal fibroblast growth factor, and vascular endothelial growth factor^11,26,27^. It also promotes the migration and proliferation of VSMCs in the coronary arteries and changes the VSMC phenotype from contractile to synthetic^27^. Through these mechanisms, nicotine accelerates the formation of the atherosclerotic characteristics of intima lesions. Our results showed that the proliferation of the tunica intima was dependent on the nicotine dose.

Nicotine injection with balloon overdilation induces atherosclerotic lesions by activating KLF4 expression. Atherosclerosis is a chronic inflammatory disease characterized by the narrowing of the coronary arteries. The coronary angiogram and OCT results in our study showed significant intimal hyperplasia of the porcine coronary arteries in the nicotine injection with balloon overdilation groups. As time passed from 4 to 8 weeks, the loss of collagen positive was observed in the imtial hyperplasia lesion. Loss of collagen due to degradation of extracellular matrix is important characteristic of necrotic core, which is important factor for plaque rupture prediction^28^. Narrowing is closely related to characteristic plaque lesions. Pathological studies have reported a large number of macrophags marker positive-cells, such as CD68, in the plaque resulting from the VSMCs, which are normally expressed in the coronary arteries^4,5^. KLF4 is an important transcription factor of VSMCs phenotypic transformations, macrophage polarization, lymphocyte differentiation, and cell proliferation as atherosclerosis progression^29–31^. In experimental studies, KLF4 expression was found to be associated with VSMC phenotypic switching. KLF4 downregulation delayed phenotypic switching of VSMCs under stressful in vitro conditions^32,33^. KLF4 knockout mice demonstrated decreased differentiation of the VSMCs to macrophage-like cells and increased plaque stability and fibrous cap thickness compared to the wild-type mice^34^. In our results, balloon overdilation or nicotine injection slightly increased the expression of KLF4 and CD68 in porcine coronary arteries. Nicotine injection with balloon overdilation significantly increased the expression of KLF4 and CD68. Immunohistochemistry results demonstrated that, CD68 positive cells co-expressed SMA and KLF4 in the intimal hyperplasia lesions. These results demonstrated that nicotine injection with balloon overdilation can induce intimal hyperplasia lesion at 4 weeks, which developed the atherosclerotic lesion at 8 weeks.

**N**icotine injection with balloon overdilation porcine model is a novel atherosclerotic model that serves as an alternative porcine model for development of coronary stents.

## Methods

### Animal and study groups

The Institutional Animal Care and Use Committee (IACUC) of Chonnam National University Hospital (IACUC approval No.CNUHIACUC-18006) approved this animal study, and it conforms to the *Guide for the Care and Use of Laboratory Animals* published by the US National Institutes of Health (NIH Publication No. 85-23, revised 1996) and the ARRIVE (Animal Research: Reporting of In Vivo Experiments) guidelines for reporting animal research^19^. Study animals were Yorkshire X Landrace F1 crossbred castrated male swine (15–20 kg) provided by Chuwol grandparent farm located in the southwest of the Republic of Korea. Study animals were randomly allocated to group 1 [negative control, n = 10], group 2 [balloon overdilation, n = 10], group 3 [0.05 mg/kg nicotine injection, n = 10], group 4 [0.25 mg/kg nicotine injection, n = 10], group 5 [0.5 mg/kg nicotine injection, n = 10], group 6 [0.05 mg/kg nicotine injection with balloon overdilation, n = 10], group 7 [0.25 mg/kg nicotine injection with balloon overdilation, n = 10], and group 8 [0.5 mg/kg nicotine injection with balloon overdilation, n = 10].

### Nicotine injection and balloon overdilation procedures

Aspirin 100 mg and clopidogrel 75 mg per day were administered to the study animals for 5 days before the procedure. On the day of procedure, pigs were anesthetized with zolazepam and tiletamine (2.5 mg/kg), xylazine (3 mg/kg) and azaperone (6 mg/kg). Continuous oxygen was supplied through an oxygen mask. After subcutaneous 2% lidocaine injection, the left carotid artery was surgically exposed, and a 7-French (Fr) sheath was inserted. Continuous hemodynamic and surface electrocardiographic monitoring was maintained throughout the procedure. Subsequently 5,000 units of heparin was administered intravenously as a bolus and the target coronary artery was engaged using standard 7-Fr guide catheters and baseline angiograms were performed using nonionic contrast agent in two orthogonal views. Balloon overdilation was performed by inflating the balloon with the resulting balloon-to-artery diameter ratio in the range of 1.3–1.4:1 to achieve maximum luminal patency. Coronary angiograms were obtained immediately after balloon overdilation. After the balloon overdilation procedures, the pigs were administered 0.05 mg/kg, 0.25 mg/kg, and 0.5 mg/kg nicotine via intramuscular routes daily for one month. One month later, the pigs underwent repeat angiography in the same orthogonal views and were euthanized with an intracoronary injection of potassium chloride (15%, 20 mL) (Supplementary Fig. S1). The samples of the coronary arteries were pressure-perfusion fixed at 70 mmHg in 10% neutral buffered formalin for 24–48 h.

### Optical coherence tomography analysis

Optical coherence tomography (OCT) was performed using a 2.7 Fr C7 Dragonfly Optis Imaging Catheter (LightLab Imaging, Inc.). The catheter was placed in the distal native artery to the balloon overdilated lesion, and automatic pullback at a speed of 20 mm/s was done during continuous automatic flushing of iodixanol at the rate of 2–5 mL/s using a Medrad injector (Medrad Inc.). Qualitative and quantitative measurements were achieved using OCT to assess the balloon overdilation area at one-month follow-up. OCT images were analyzed at a 1 mm distance in a blinded fashion. Volumetric analysis was performed to measure the lumen area and intimal hyperplasia area.

### Histopathologic and immunohistochemical analyses

The fixed coronary artery samples were placed perpendicular to the direction of blood flow at 5 mm intervals and were embedded in paraffin. The coronary artery samples were sectioned at a thickness of 5 µm on a rotary microtome for histopathologic and immunohistochemical analyses. Hematoxylin and eosin staining and picrosirius red staining were performed for the histopathologic analysis. Immunohistochemical analysis was carried out to identify the proteins expression. Non-specific reactivity was blocked with 3% fetal bovine serum in phosphate-buffered saline for 60 min. Immunohistochemistry was performed using anti-rabbit monoclonal alpha smooth muscle actin (SMA, 1:100; Abcam), anti-rabbit polyclonal Krüppel-like factor 4(KLF4, 1:100; LSBio), and anti-mouse monoclonal cluster of differentiation 68 (CD68, 1:20; Invitrogen) antibodies. The secondary antibodies were streptavidin AlexaFluor594-conjugated anti-mouse immunoglobulin G (IgG) (1:400; Invitrogen) and streptavidin AlexaFluor488-conjugated anti-rabbit IgG (1:400; Invitrogen) for fluorescence microscopy. Images were captured using Nikon eclipse 80i fluorescence microscopy.

### Western blot analysis

The porcine coronary arteries (negative control, balloon overdilation, 0.25 mg/kg nicotine injection, and 0.25 mg/kg nicotine injection with balloon overdilation) were collected to evaluate changes in protein expression. Samples were washed with cold phosphate-buffered saline and total cell lysates were prepared using lysis buffer (1 M Tris–HCl [pH 8.0], 5 M NaCl, 1% NaN_3_, 10% sodium dodecyl sulfate, 10% NP-40, and 0.5% C_24_H_39_NaO_4_). Equal sample amounts were loaded onto a 10% polyacrylamide gel. Following electrophoresis, proteins were transferred to polyvinylidene membranes. Non-specific binding was blocked with 5% skim milk in 0.1% Tween 20 for 30 min. The membranes were incubated with anti-rabbit polyclonal KLF4 (1:1000; LSBio), anti-mouse monoclonal CD68 (1:500; Invitrogen), and anti-rabbit polyclonal glyceraldehyde 3-phosphate dehydrogenase (GAPDH, 1:1,000; Santa Cruz Biotechnology, Inc.) antibodies overnight at 4 °C. After washing five times, the membranes were incubated with horseradish peroxidase-conjugated secondary antibodies, and proteins were detected using an enhanced chemiluminescence reagent (Anigen). Densitometry was performed using the Image J software (https://imagej.net/), and statistical analysis was carried out using Prism 5.0 (GraphPad).

### Statistical analysis

Data are represented as means ± standard deviations of at least three independent experiments. Statistical differences were tested by ANOVA with Tukey’s multiple comparison post hoc test as appropriate using Prism 5.0 (GraphPad). *P* values less than 0.05 were considered statistically significant.

## Supplementary Information

Supplementary Legends.

Supplementary Figure 1.

Supplementary Figure 2.

Supplementary Figure 3.

Supplementary Figure 4.
