# Supplementary material for: A model of atherosclerosis using nicotine with balloon overdilation in a porcine
Source: Sci Rep. 2021 Jul 1;11:13695. doi: 10.1038/s41598-021-93229-1 (PMC8249376; doi:10.1038/s41598-021-93229-1)
Supplement: Supplementary file 1 — Supplementary Legends. [file 41598_2021_93229_MOESM1_ESM.docx]

**A model of atherosclerosis using nicotine with balloon overdilation in a porcine**

Munki Kim ^1^, Han Byul Kim ^1^, Dae Sung Park ^1,2^, Kyung Hoon Cho ^3^, Dae Young Hyun ^3^, Hae Jin Kee ^1^, Young Joon Hong ^3^, Myung Ho Jeong ^1,2,3,*^.

^1^ The Cardiovascular Convergence Research Center of Chonnam National University Hospital Designated by Korea Ministry of Health and Welfare, Gwangju 61469, Republic of Korea

^2^ Korea Cardiovascular Stent Research Institute, Jangsung 57248, Republic of Korea

^3^ Division of Cardiology of Chonnam National University Hospital, Cardiovascular Convergence Research Center Nominated by Korea Ministry of Health and Welfare, Gwangju, Republic of Korea

Address Correspondence to:

Myung Ho Jeong

The Cardiovascular Convergence Research Center of Chonnam National University Hospital Designated by Korea Ministry of Health and Welfare

Gwangju 61469, Republic of Korea

Tel : +82-62-220-6243

[myungho@chollian.net](mailto:myungho@chollian.net)

**Supplementary Information**

**Coronary angiographic findings**

Coronary angiography, the gold standard for assessing coronary artery diseases, was performed to identify the changes occurring in the porcine coronary arteries at four weeks after the experiment. The left anterior descending artery and left circumflex artery (LCX) were observed on baseline coronary angiography (Supplementary Fig.1A, B). A coronary balloon was placed in the LCX and inflated to induce a mechanical injury to the vessel walls (Supplementary Fig.1C, D). Four weeks after the experiment, the simple balloon overdilation group showed no angiographic lesions (Supplementary Fig. 1E). However, the 0.25 mg/kg nicotine injection with balloon overdilation group exhibited significant narrowing of the coronary arteries in the injured LCX (Supplementary Fig. 1F).

**Histopathologic changes following nicotine with balloon overdilation**

All nicotine injection dose groups showed changes in the lumen area, area of media, and area of intimal hyperplasia. Lumen area did not show the significant changes between three groups (Supplementary Fig. 2A-C). Area of tunica media significantly increased in the 0.25mg/kg nicotine injection and 0.5mg/kg nicotine injection groups compared to the 0.05mg/kg nicotine injection groups (4.27 ± 2.04 vs 6.88 ± 3.1 vs 7.8 ± 2.58). The 0.25mg/kg nicotine injection groups did not show statistical significances compared with the 0.5mg/kg nicotine injection group. The area of intimal hyperplasia significantly increased in the 0.25mg/kg nicotine injection and 0.5mg/kg nicotine injection groups compared to 0.05mg/kg nicotine injection groups (1.14 ± 0.89 vs 3.83 ± 1.98 vs 4.44 ± 2.43). The 0.25mg/kg nicotine injection groups did not show statistical significance in comparison with the 0.5mg/kg nicotine injection group.

**CD68 expression in the coronary arteries**

To identify the expression of CD68 positive cells, immunohistochemistry was performed. CD68 positive cells were not observed in the control groups (Supplementary Fig. 3A-C). Balloon overdilation group showed intimal hyperplasia, but CD68 positive cells were not observed (Supplementary Fig. 3D-F). 0.05mg/kg nicotine groups did not showed CD68 positive cells (Supplementary Fig. 3G-I). However, 0.25 and 0.5mg/kg Nicotine treatment group showed CD68 positive on the tunica itima area (Supplementary Fig. 3J-O). For quantification, CD68 and DAPI positive cells were counted using ImageJ. The ratio of CD68/DAPI significantly increased in 0.25mg/kg injection and 0.5mg/kg injection groups compared to the 0.05mg/kg nicotine injection groups (27.90 ± 5.62 vs 52.09 ± 8.74 vs 60.36 ± 9.86). The 0.25mg/kg nicotine injection groups did not show a statistically significant difference with the 0.5mg/kg nicotine injection groups. (Supplementary Fig. 3P-S)

**Western blot full length images**

CD68 molecular weight : 110 kD, KLF4 molecular weight : 62kD, GAPDH molecular weight 37 kD.

**Supplementary data figure legends**

**Supplementary Figure 1. Coronary angiographic findings.** (A) Base line image shows the distribution of the left anterior descending and left circumflex (LCX) arteries. (B) The balloon overdilation was induced using over inflation of a coronary balloon. (C) One month after the injury, angiography showed the LCX diameter was similar to that at base line. (D-E) After the balloon overdilation, nicotine was administered via intramuscular injection. (F) One month later, angiography showed a clearly narrowed LCX.

**Supplementary Figure 2. Histopathologic changes following nicotine with balloon overdilation.** Lumen area did not show significant changes between the three groups (Supplementary Fig. 2A-C). Area of the tunica media significantly increased in the 0.25 mg/kg nicotine injection and 0.5 mg/kg nicotine injection groups compared to the 0.05 mg/kg nicotine injection groups (4.27 ± 2.04 vs 6.88 ± 3.1 vs 7.8 ± 2.58). The 0.25 mg/kg nicotine injection groups did not show any statistically significant difference compared with the 0.5 mg/kg nicotine injection group. The area of intimal hyperplasia significantly increased in the 0.25 mg/kg nicotine injection and 0.5 mg/kg nicotine injection groups compared to the 0.05 mg/kg nicotine injection groups (1.14 ± 0.89 vs 3.83 ± 1.98 vs 4.44 ± 2.43). The 0.25 mg/kg nicotine injection groups did not show a statistically significant difference with the 0.5 mg/kg nicotine injection group. Bar: 500 µm

**Supplementary Figure 3. CD68 expression in the coronary arteries.** To identify the expression of CD68 positive cells, immunohistochemistry was performed. CD68 positive cells were not observed in the control groups (A-C). Balloon overdilation group showed intimal hyperplasia, but CD68 positive cells were not observed (D-F). 0.05mg/kg nicotine groups did not showed CD68 positive cells (G-I). However, 0.25 and 0.5mg/kg Nicotine treatment group showed CD68 positive on the tunica itima area (J-O). For quantification, CD68 and DAPI positive cells were counted using ImageJ. The ratio of CD68/DAPI significantly increased in 0.25mg/kg injection and 0.5mg/kg injection groups compared to the 0.05mg/kg nicotine injection groups (27.90 ± 5.62 vs 52.09 ± 8.74 vs 60.36 ± 9.86). The 0.25mg/kg nicotine injection groups did not show a statistically significant difference with the 0.5mg/kg nicotine injection groups. (P-S)

**Supplementary Figure 4. Western blot full length images.** CD68 molecular weight : 110 kD, KLF4 molecular weight : 62kD, GAPDH molecular weight 37 kD.

**
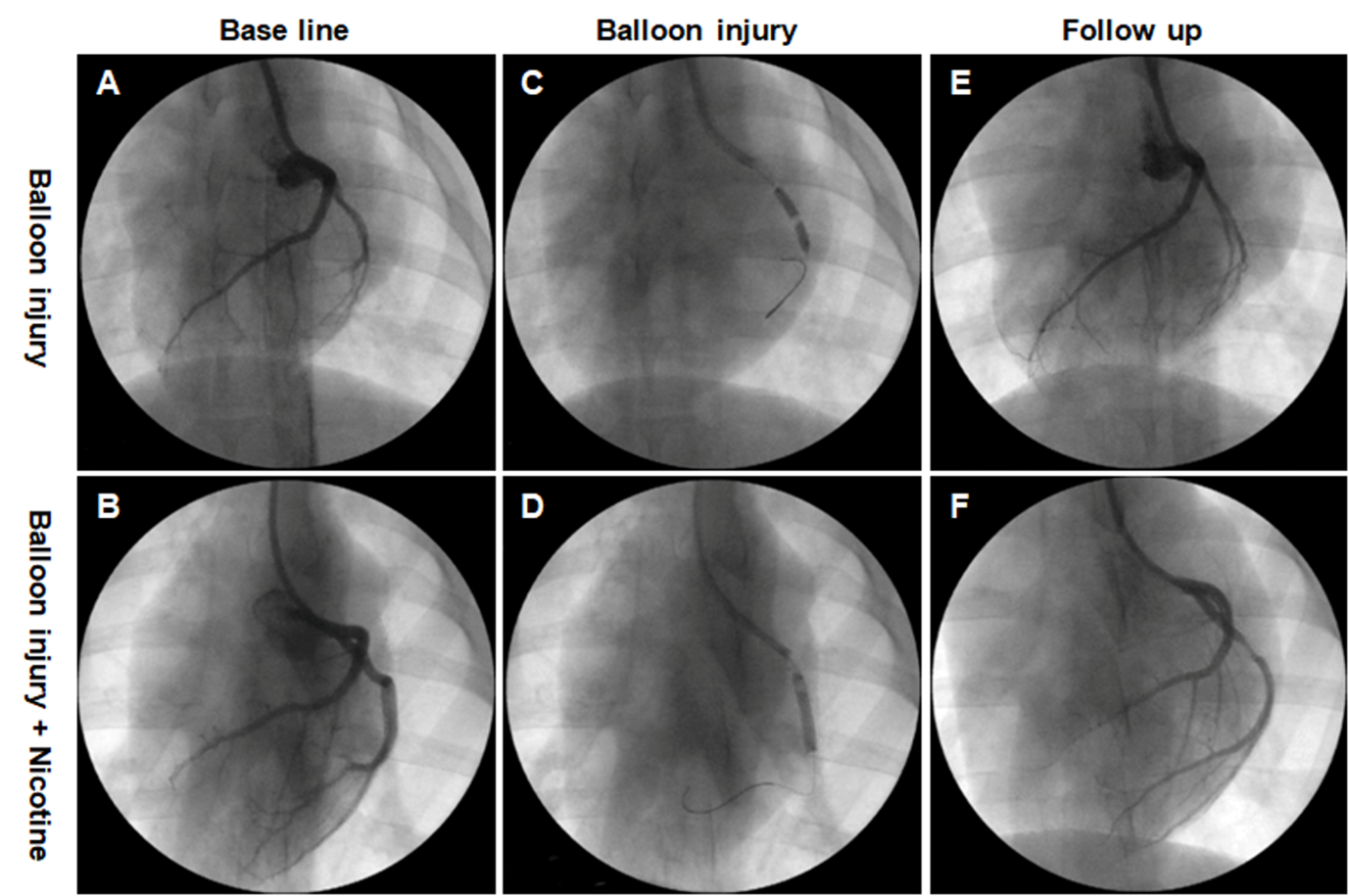
**

**Supplementary Figure 1.**


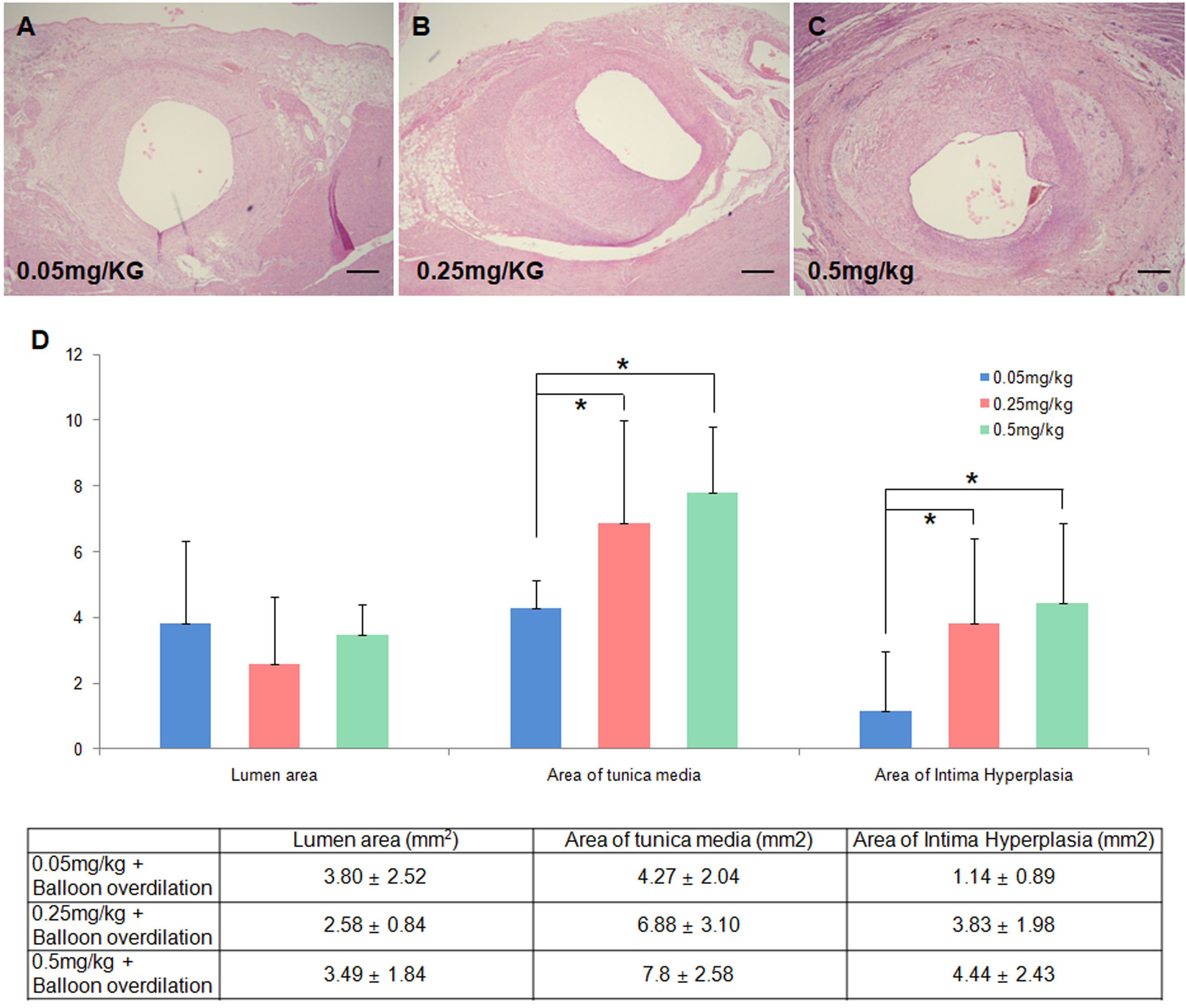


**Supplementary Figure 2.**


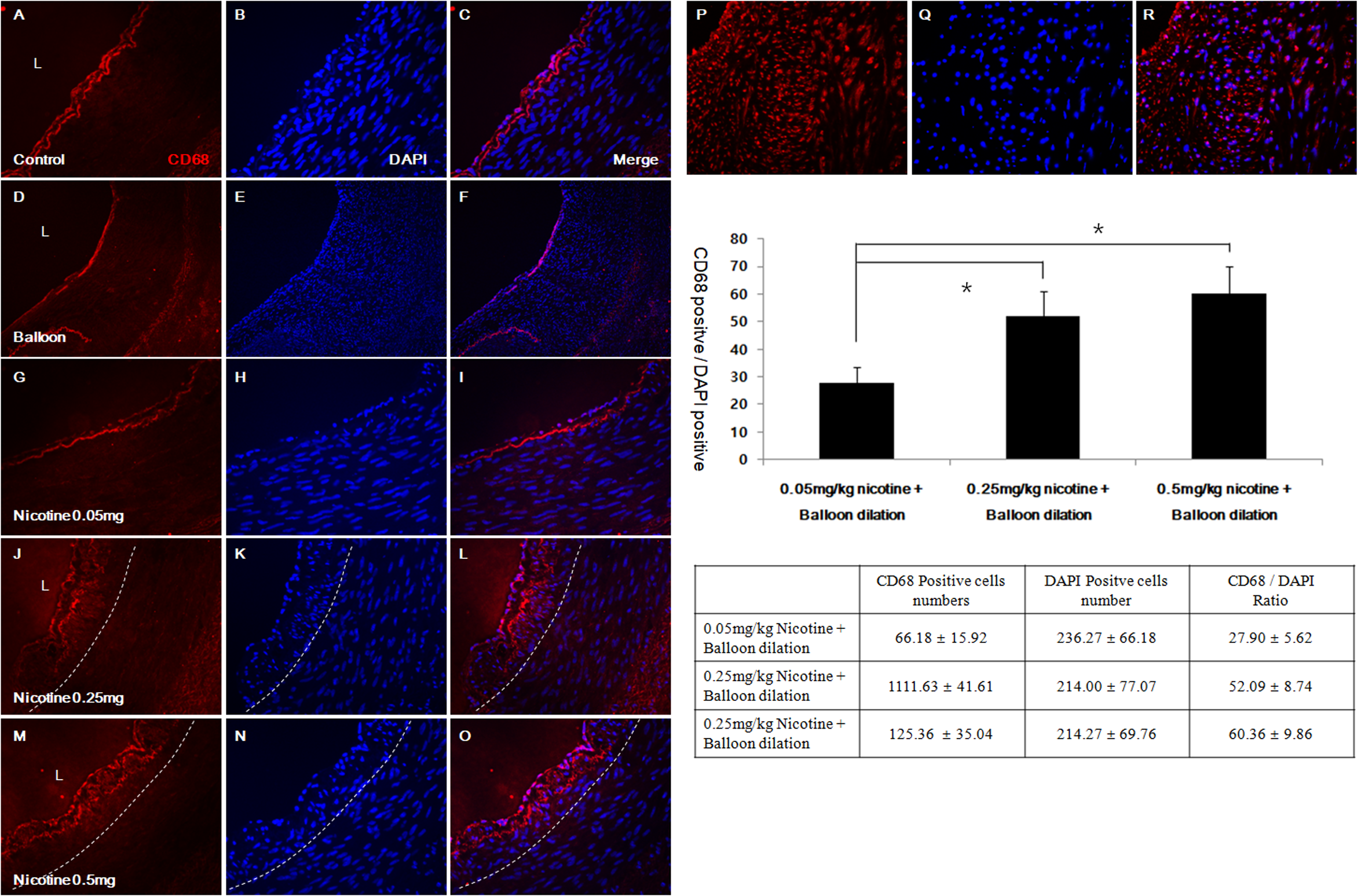


**Supplementary Figure 3.**


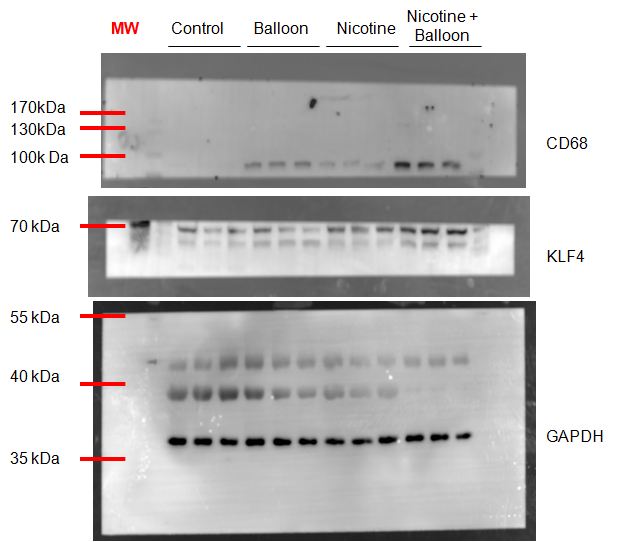


**Supplementary Figure 4.**
