# Supplementary figures and images for: A model of atherosclerosis using nicotine with balloon overdilation in a porcine
Source: Sci Rep. 2021 Jul 1;11:13695. doi: 10.1038/s41598-021-93229-1 (PMC8249376; doi:10.1038/s41598-021-93229-1)

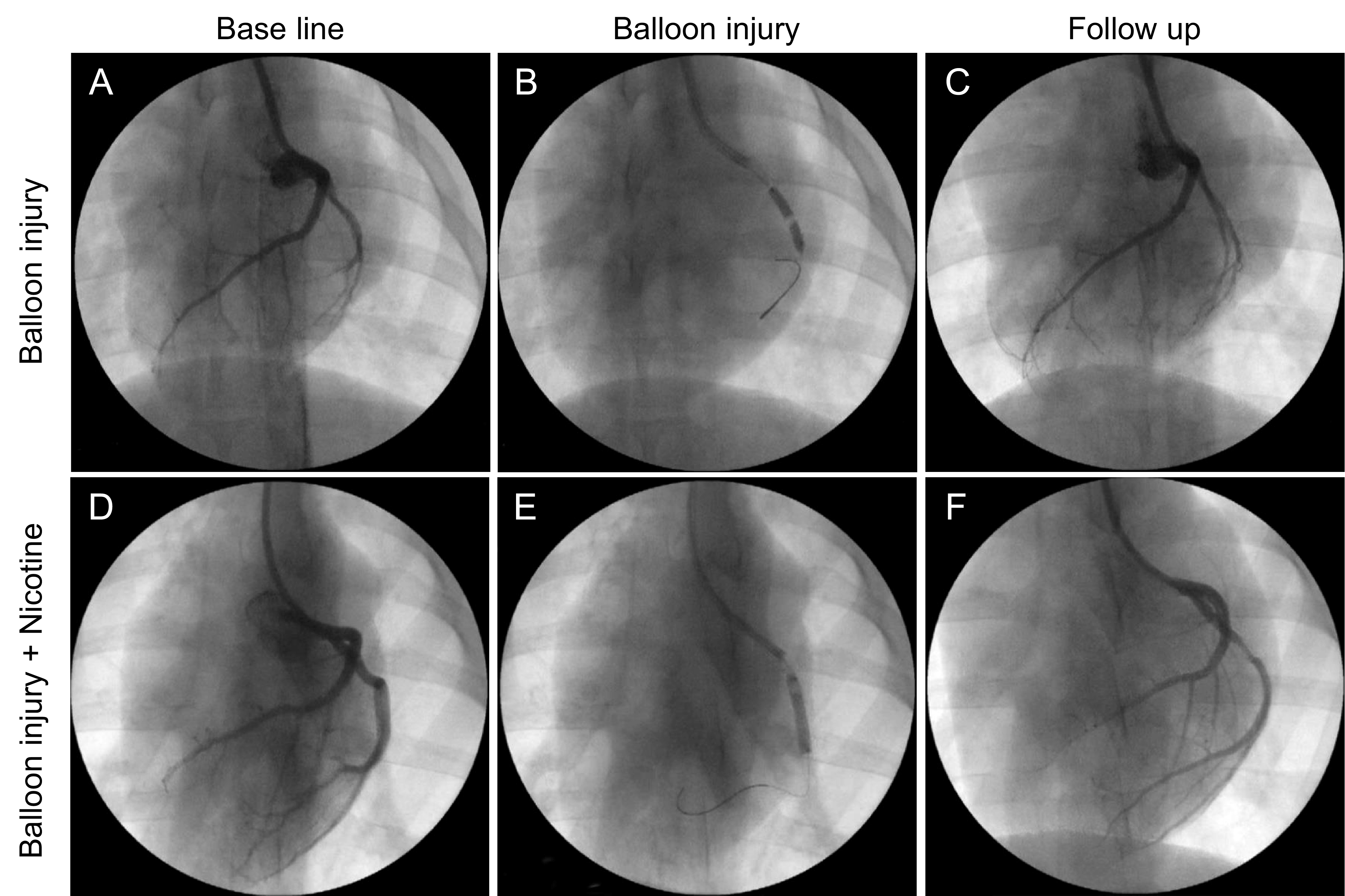

Supplement: Supplementary file 2 — Supplementary Figure 1. [file 41598_2021_93229_MOESM2_ESM.tif]

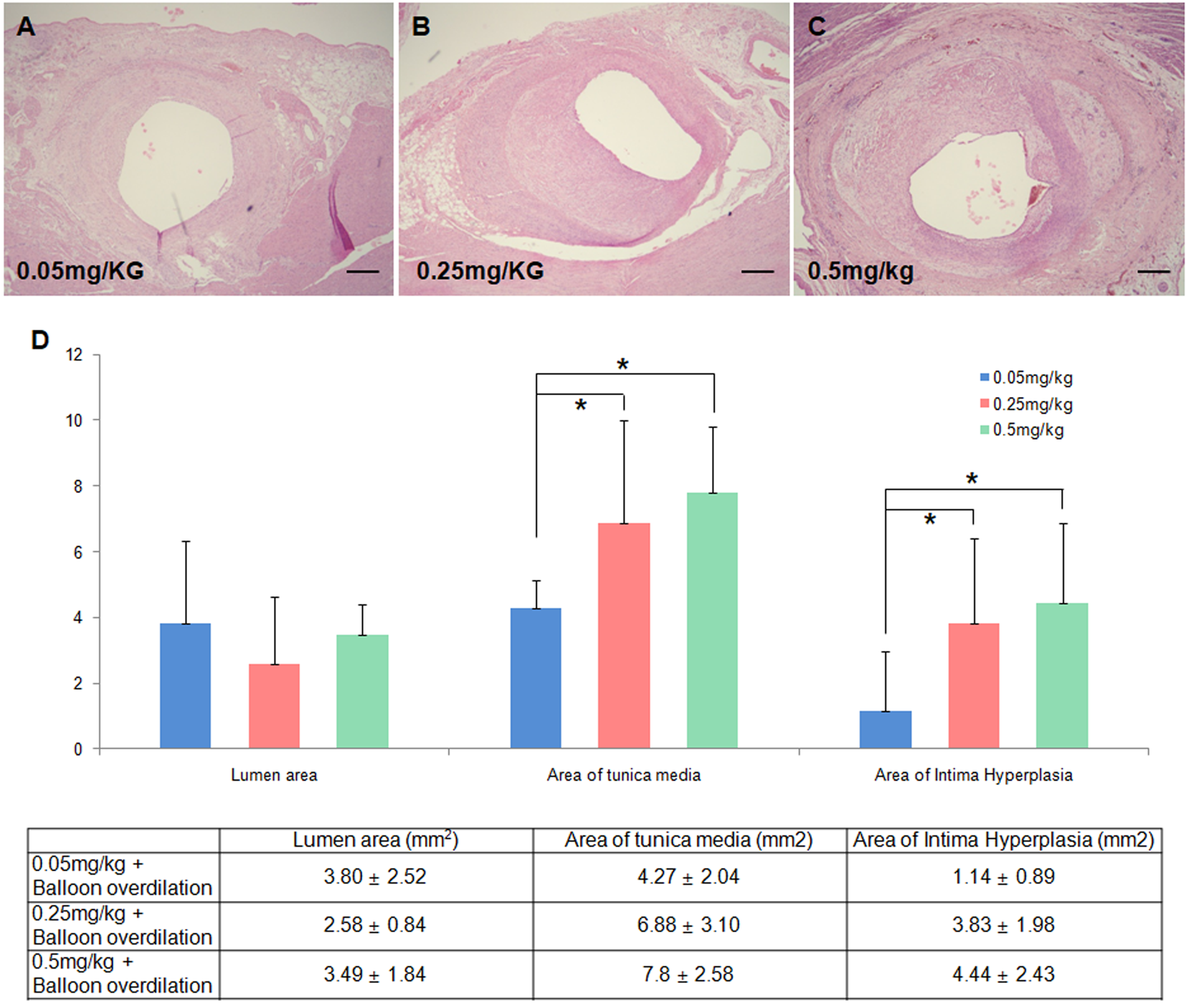

Supplement: Supplementary file 3 — Supplementary Figure 2. [file 41598_2021_93229_MOESM3_ESM.tif]

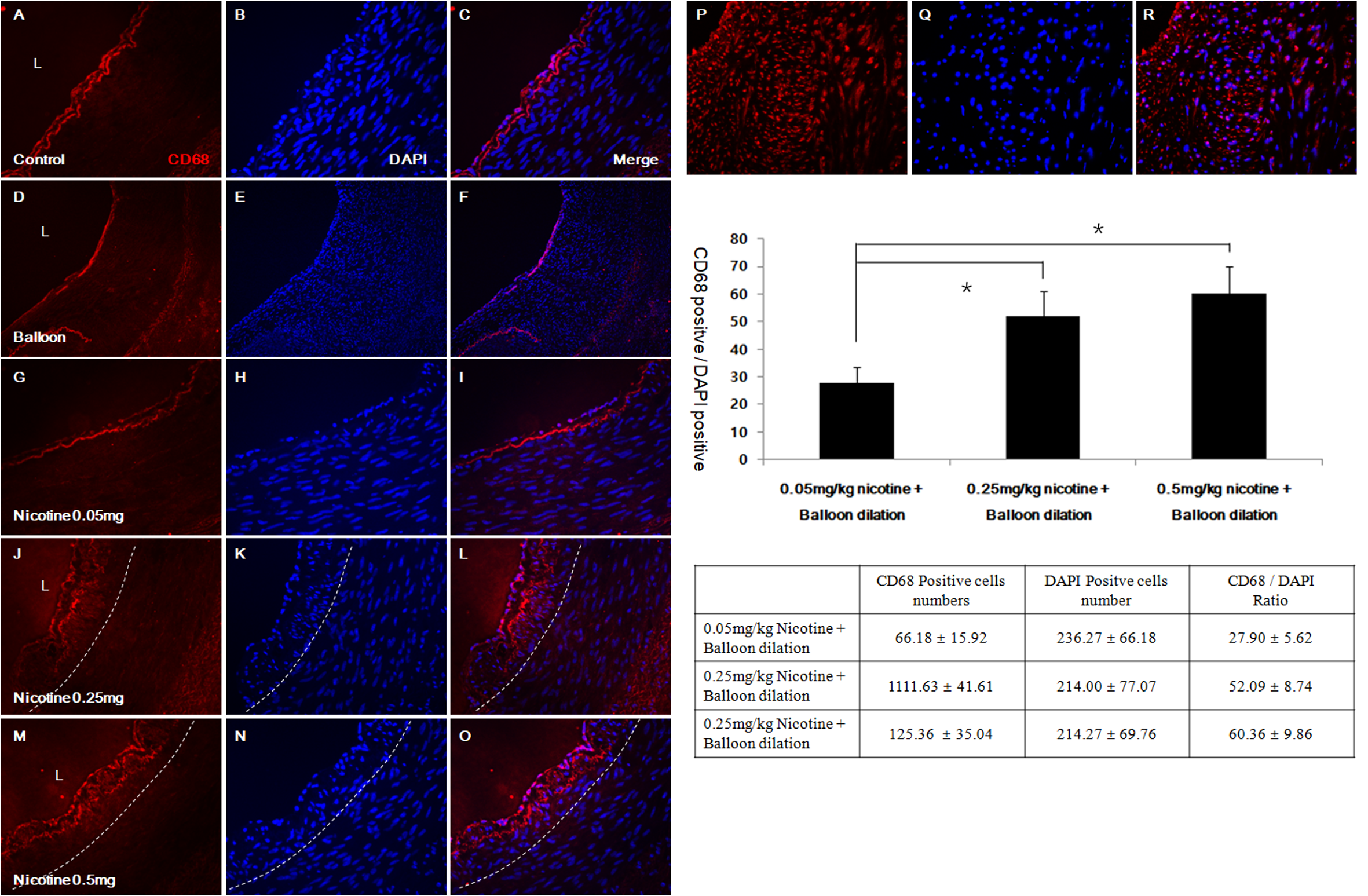

Supplement: Supplementary file 4 — Supplementary Figure 3. [file 41598_2021_93229_MOESM4_ESM.tif]

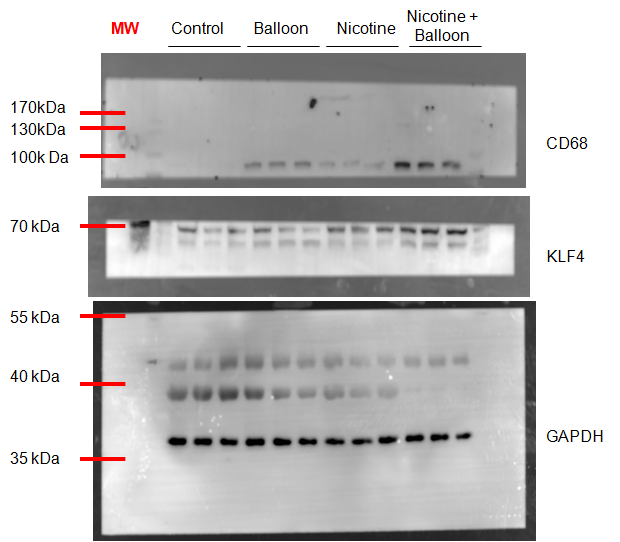

Supplement: Supplementary file 5 — Supplementary Figure 4. [file 41598_2021_93229_MOESM5_ESM.tif]
